# The Blood Neutrophil to Lymphocyte Ratio Correlates with Clinical Status in Children with Cystic Fibrosis: A Retrospective Study

**DOI:** 10.1371/journal.pone.0077420

**Published:** 2013-10-02

**Authors:** Catherine E. O’Brien, Elvin T. Price

**Affiliations:** 1 University of Arkansas for Medical Sciences College of Pharmacy, Department of Pharmacy Practice, Little Rock, Arkansas, United States of America; 2 University of Arkansas for Medical Sciences College of Medicine, Department of Pediatrics, Division of Pharmacology and Toxicology Little Rock, Arkansas United States; 3 University of Arkansas for Medical Sciences College of Pharmacy, Department of Pharmaceutical Sciences Little Rock, Arkansas United States; University of Tübingen, Germany

## Abstract

**Purpose:**

The blood neutrophil to lymphocyte ratio (NLR) has been identified as a potentially useful marker of clinical outcome in disease states with an inflammatory component. The objective of this study was to evaluate the relationship between NLR and clinical status in children with cystic fibrosis.

**Methods:**

This was a retrospective chart review. Data collected included NLR, body mass index, and forced expiratory volume in 1 second (FEV1) while asymptomatic, and during hospitalizations for pulmonary exacerbation. An NLR breakpoint of 3 was used for comparisons of body mass index and FEV1.

**Results:**

A total of 159 charts were reviewed. An NLR ≥ 3 was significantly associated with lower body mass index and lower FEV1. NLR during hospitalization was significantly higher than NLR while asymptomatic. NLR measured during the first 3 months of life was negatively correlated with FEV1 at age 12.

**Conclusion:**

NLR correlates with clinical status in children with cystic fibrosis and may be a useful biomarker in this population.

## Introduction

Cystic fibrosis (CF) is an autosomal recessive genetic disease that affects approximately 30,000 people in the United States [1]. Patients develop chronic pulmonary infection with various organisms, notably *Pseudomonas aeruginosa*, and neutrophil-dominated inflammation plays a role in the pathophysiology [2-5]. Over time, inflammation causes tissue damage and contributes to progressive lung function decline. Irreversible changes can be seen on chest radiography before 2 years of age [6], but other measures of early lung damage are lacking. The standard for monitoring disease progression and identifying pulmonary exacerbations is pulmonary function testing (PFTs) and forced expiratory volume in 1 second (FEV1) is the current clinical endpoint. Children are generally not able to perform PFTs reliably and consistently until the age of 5-6 years, and clinicians rely on clinical signs and symptoms to gauge whether more aggressive pulmonary treatments are necessary.

The blood neutrophil to lymphocyte ratio (NLR) has been identified as a potentially useful marker of inflammation that is prognostic of outcome in various disease states, including cardiovascular disease and gastrointestinal cancers [7-11]. As inflammation in the CF lung is neutrophil-dominated, NLR may be a useful biomarker of clinical status in children with CF. To date, there have been no reports describing the relationship of this biomarker to clinical status in people with CF. NLR, which is calculated from the complete blood count, is an easily accessible biomarker; it does not require specialized equipment or assays. If it proves useful as a biomarker in children with CF, it could be easily implemented. Here we describe a retrospective pilot study designed to determine whether this may be a useful biomarker for further study in the CF population. Our primary aim was to evaluate the relationship between blood NLR and clinical status in children with CF.

## Results

A total of 159 charts were reviewed. Of these, 126 subjects had a clinic visit with a corresponding CBC while clinically stable. There were 37 subjects hospitalized for a pulmonary exacerbation with a CBC measured within 2 days of admission. 14 subjects had an NLR of ≥ 3 while clinically stable. The age range for subjects with an NLR ≥ 3 was 9-18 years, and so only subjects 9 years of age or older were included for this analysis. Clinical measures compared by NLR breakpoint are presented in Tables 1 and 2. Subjects with an NLR ≥3 had statistically significantly lower FEV1 and BMI percentile compared to those with an NLR < 3. The mean age did not differ between those with an NLR ≥ 3 and those with an NLR ˂ 3.

**Table 1:** FEV1 percent of predicted compared by NLR breakpoint of 3 in children 9-18 years of age.

	NLR < 3	NLR ≥ 3	
Clinical Parameter	N = 56	N = 14	P-value
FEV1 % of predicted	97.2 ± 15.6	84.1 ± 15.1	0.006

Abbreviations: NLR − neutrophil to lymphocyte ratio, FEV1 − forced expiratory volume in 1 second

**Table 2:** BMI percentile compared by NLR breakpoint of 3 in children 9-18 years of age.

	NLR < 3	NLR ≥ 3	
Clinical Parameter	N = 60	N = 14	P-value
BMI percentile	53.5 ± 25.0	33.4 ± 26.5	0.009

Abbreviations: NLR − neutrophil to lymphocyte ratio, BMI − body mass index

**Figure 1:**
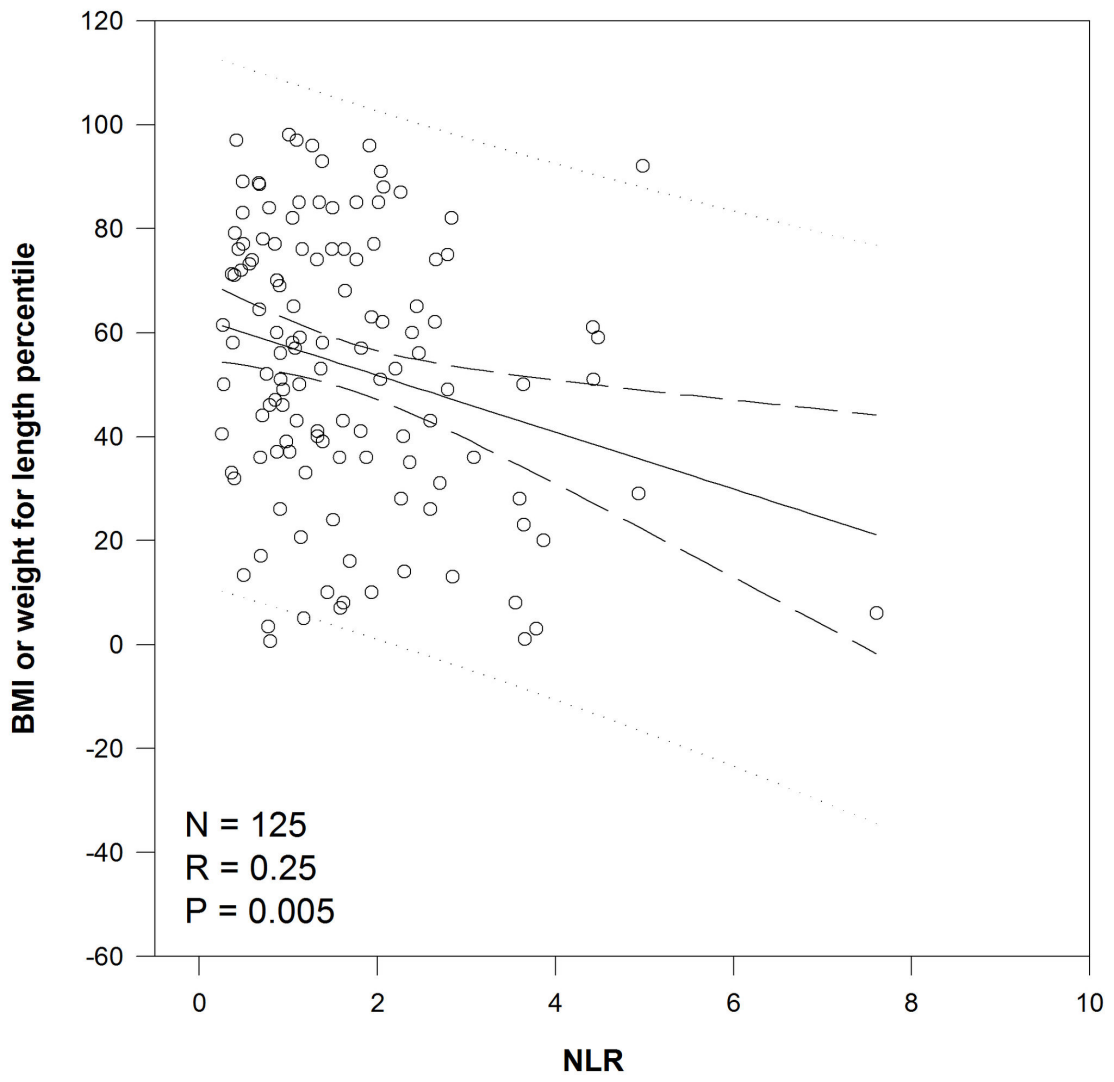
Correlation between NLR and nutritional status. Linear regression analysis of the relationship between NLR and nutritional status as measured by BMI or weight for length percentile. Abbreviations: BMI – body mass index, NLR – neutrophil to lymphocyte ratio.

**Figure 2:**
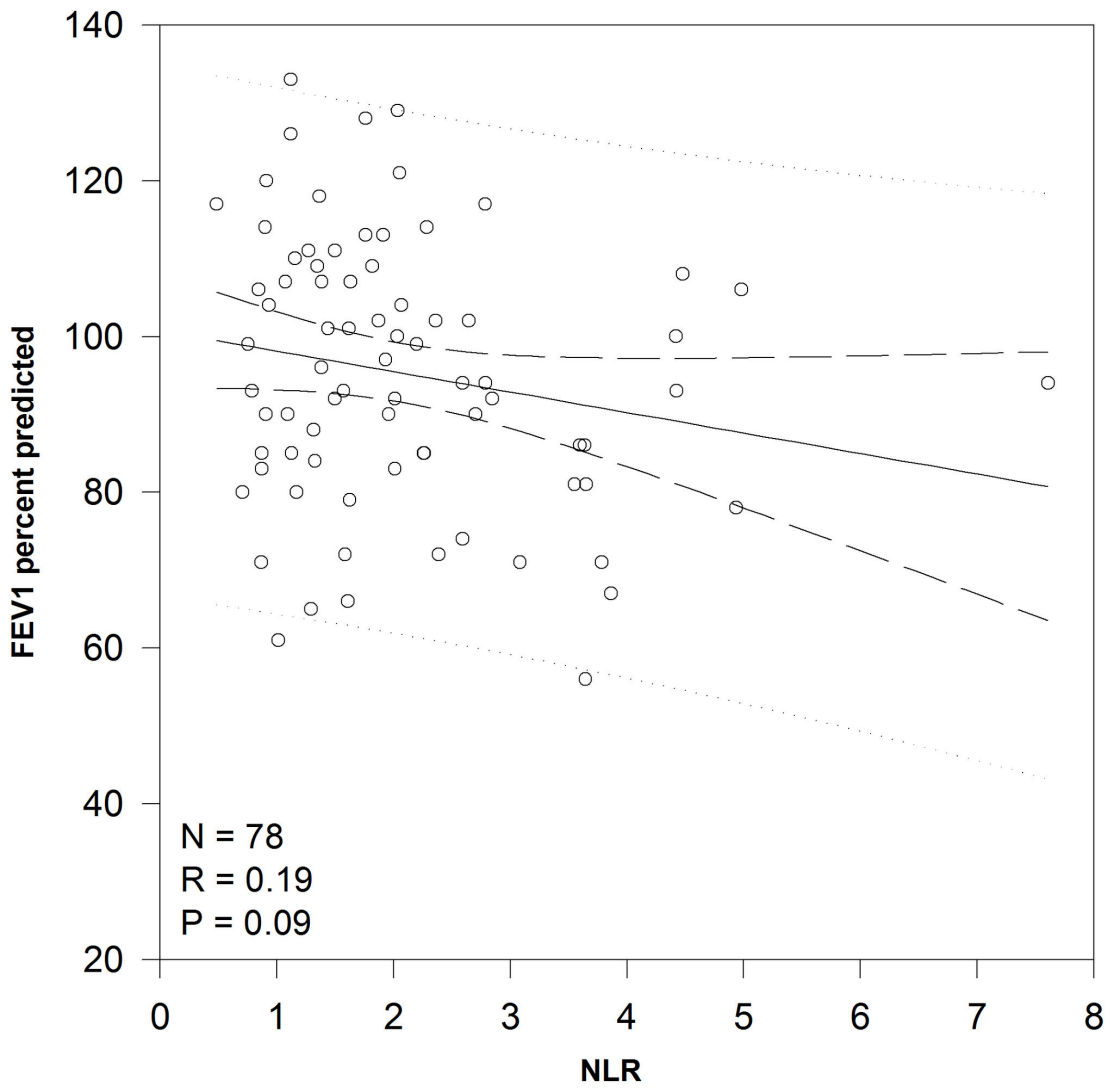
Correlation between NLR and lung function. Linear regression analysis of the relationship between NLR and lung function as measured by FEV1 percent predicted. Abbreviations: FEV1 – forced expiratory volume in one second, NLR – neutrophil to lymphocyte ratio.

**Figure 3:**
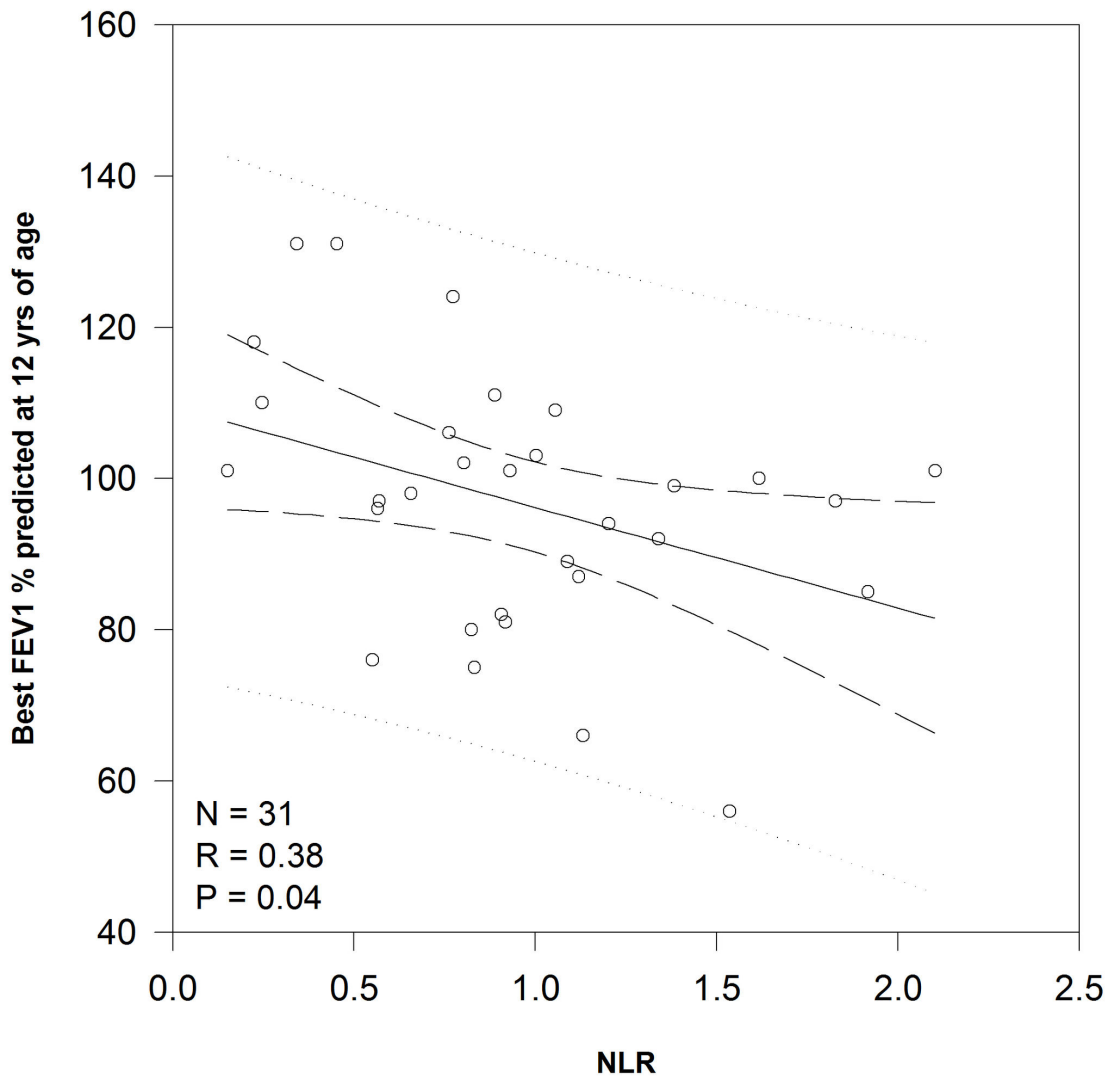
Correlation between NLR ratio during first 36 months of life and lung function at 12 years of age. Linear regression analysis of the relationship between NLR measured during early childhood and later lung function as measured by FEV1 percent predicted at 12 years of age. Abbreviations: FEV1 – forced expiratory volume in one second, NLR – neutrophil to lymphocyte ratio.

Linear regression demonstrated a significant relationship between NLR and nutritional status (Figure 1) with each increase of 1 in NLR correlating to a decrease of 5.5 percentile points for BMI/weight for length (P=0.005). There was a trend (P=0.09) toward a significant relationship between NLR and FEV1 (Figure 2) with a decrease of 2.6 percentage points for each increase in NLR of 1. The relationship between NLR measured during the first 36 months of life and later lung function was more robust. While there was not a significant relationship between NLR and FEV1 measured at 7 years of age, NLR explained 14.5% of FEV1 measured at 12 years of age (P=0.035). For each increase in NLR of 1, there was a decrease in FEV1 of 13.3 percentage points (Figure 3).

Among the 37 subjects (mean age of 11.3±5.0) who had NLR measured both while clinically stable and during the first 2 days of hospitalization for a pulmonary exacerbation, there were 51 hospital admissions for pulmonary exacerbation. NLR during a pulmonary exacerbation was significantly elevated compared to NLR measured while clinically stable (2.8±2.1 versus 1.9±1.1, respectively, P=0.007).

## Discussion

NLR is significantly correlated with clinical status in children with CF. Children with an NLR ≥ 3 have significantly worse lung function and nutritional status. According to lung function stratification referred to in the CF pulmonary guidelines [16] and commonly used in clinical trials, the means for FEV1 suggest that children with NLR ≥ 3 have mildly impaired lung function (FEV1 70-90% of predicted) compared to normal lung function (FEV1 ≥ 90% of predicted) in children with NLR < 3. This difference is likely to widen as these children age and is consistent with studies that have shown a correlation between elevated sputum levels of neutrophilic chemokines and decreased pulmonary function as measured by FEV1 [4]. Nutritional status is an important clinical measure in children with CF as it directly correlates with lung function; the nutritional goal for children with CF is a BMI percentile of greater than the 50^th^ [1]. In this group of children with CF, the children with NLR < 3 had a mean BMI percentile at goal, while those with NLR ≥ 3 did not.

In several other disease states with an inflammatory component, an elevated NLR has been implicated as a marker of poorer outcome. Patients undergoing resection for non-small cell lung cancer had a worse 5-year survival rate if their preoperative NLR was ≥ 2.5 [15]. Among adults with stage 4 chronic kidney disease, those with an NLR < 3 had a slower progression of their disease [13]. In patients with metastatic renal cell carcinoma treated with sunitinib, pretreatment NLR ≤ 3 was associated with better overall and progression-free survival [12]. Among patients with clear cell renal cell carcinoma undergoing resection, a preoperative NLR ≥ 2.7 was associated with a lower 10-year recurrence-free survival [14]. In patients receiving elective major vascular surgery, a preoperative NLR > 5 was associated with decreased survival at 2 years [17]. A preoperative NLR > 4 has also been associated with recurrence of colon cancer after curative resection [18], and an NLR ≥ 5 with decreased overall survival after colectomy [19]. Patients undergoing surgical resection for gastric cancer also experienced worse survival if their preoperative NLR was ≥ 4 [10]. In a study of patients with metastatic colorectal cancer undergoing chemotherapy, an NLR > 5 was an independent predictor of overall survival [20]. The investigators also found that when NLR normalized after a round of chemotherapy, this was associated with better outcome. An elevated NLR has also been associated with adverse outcomes in cardiovascular disease. Patients with acute decompensated heart failure in the highest tertile for NLR experienced increased mortality and 30-day readmission rate. Patients in this study in the highest tertile had a mean NLR of 9.6 [11]. An elevated NLR has also been associated with increased mortality after both non-ST-elevation and ST-elevation myocardial infarction [9,21]. Finally, an elevated NLR has been suggested as an adjunct to clinical evaluation in the diagnosis of acute appendicitis, with a better prognostic value than C-reactive protein or white blood cell count [22].

Other biomarkers of inflammation in peripheral blood have been evaluated in patients with CF, notably C-reactive protein (CRP) and neutrophilic chemokines such as IL-8. CRP has been associated with clinical status in people with CF, but it is a general biomarker of inflammation and does not differentiate between inflammation driven by neutrophils versus other origins, such as eosinophils as in allergic inflammation. It is also produced in response to inflammation and thus an elevation in CRP is likely to be a later event than elevation in NLR. Studies have been conducted evaluating the correlation of neutrophilic chemokines to clinical outcome in patients with CF and the results are conflicting. Biomarkers in expectorated or induced sputum have also been evaluated for their utility in predicting disease severity as measured by FEV1; most notably IL-8, neutrophil count, and neutrophil elastase. Coefficients of correlation vary between studies, with a range of -0.28 to -0.72 for IL-8, -0.29 to -0.57 for neutrophil count, and -0.35 to -0.75 for neutrophil elastase [23-25]. NLR is an easy biomarker to collect and measure and is not associated with the same methodological challenges as the collection of induced sputum. Collecting expectorated sputum is not invasive, but is difficult to collect from young patients who tend to swallow expectorated sputum.

Future studies should evaluate whether the associations between NLR and clinical status persist into adulthood. It would also be important to establish whether the NLR changes in response to pharmacotherapy and if this results in a change in clinical status.

### Limitations

This was a small retrospective study and thus has the associated limitations. The correlations with lung function and nutritional status are from cross-sectional data, and thus no conclusions can be drawn regarding how the timing of an elevated NLR is related to clinical status. Markers of inflammation are not routinely measured at our institution at clinic visits or during hospitalization, and thus were not available to compare to NLR. A larger prospective study is necessary to establish the utility of NLR as a biomarker in children with CF and the appropriate breakpoint. It is also challenging to evaluate its usefulness in young children with CF who are unable to perform pulmonary function testing. Finally, there has not been a methodical investigation to establish a reference range for NLR in healthy subjects and current studies evaluating the utility of this marker in various disease states have determined breakpoints based on a comparison of outcomes. It is possible that results would be different if compared to a healthy cohort.

## Methods

This retrospective study was approved by the University of Arkansas for Medical Sciences Institutional Review Board. Informed consent was waived by this same Institutional Review Board as this was a retrospective study and all data collected was preexisting.

This was a retrospective chart review of all pediatric CF patients followed at the Arkansas CF Center. Subjects were eligible if they had a diagnosis of CF and at least one hospitalization for pulmonary exacerbation or one clinic visit between October 2010 and October 2011 with a corresponding complete blood count (CBC). Subjects had to be clinically stable at the clinic visit (i.e. no diagnosis of a pulmonary exacerbation). The CBC during the hospitalization had to be within 2 days of admission.

Data collected included general demographics, CBC at the clinic visit and the first CBC measured during the first 3 years of life, lung function (as measured by FEV1 percent of predicted when compared to established reference values) at the clinic visit and at 7 and 12 years of age, nutritional status (as measured by body mass index (BMI) percentile) at the clinic visit, and CBC measured during the first 2 days of hospitalization for pulmonary exacerbations. If patients were diagnosed with a pulmonary exacerbation at the clinic visit, they were not considered “clinically stable” and the CBC with corresponding BMI and FEV1 were not collected.

The first objective of the study was to evaluate the relationship of NLR measured while clinically stable (i.e. at the clinic visit) to clinical status. Clinical status was evaluated by two measures: nutritional status as measured by BMI percentile (weight-for-length percentile was used for subjects less than 2 years old) and FEV1. The second objective was to compare NLR while clinically stable to NLR upon hospitalization for a pulmonary exacerbation. The third objective was to explore the possible utility of NLR as a predictor of later outcome. For this objective, the relationship between NLR measured during early childhood (the first 3 years of life) was compared to FEV1 at 7 and 12 years of age. The age of 7 was chosen because younger children are frequently unable to reliably and consistently perform pulmonary function testing until approximately 6 years of age. The age of 12 was chosen because it provided the best likelihood of having access to both an early CBC and a later FEV1 in the electronic medical record.

### Data handling and analysis

NLR was calculated from values collected from the CBC by dividing the absolute neutrophil count (both segs and bands) by the absolute lymphocyte count.

Descriptive statistics were used to describe the study population. A breakpoint of 3 for the NLR was used to compare clinical measures FEV1, BMI/weight-for-length, and exacerbation frequency. As this was a pilot study, there was no preliminary data in CF subjects from which to choose an appropriate breakpoint for comparison of measures of clinical status. A breakpoint of 2.5 is the lowest published breakpoint that is significantly associated with clinical outcome [12-15]. We also had a relatively small number of subjects with an NLR ≥ 3 and analysis would have been limited with a higher breakpoint. Subjects were age-matched for these comparisons and t-tests or Mann-Whitney rank-sum were used as appropriate. Linear regression was used to test for correlations between NLR and clinical status as measured by FEV1 and nutritional status. NLR measured during a period of clinical stability was compared to serum NLR measured during the first 2 days of hospitalization for a pulmonary exacerbation for the same patients. Wilcoxon signed-rank test was used for this comparison. A statistical software package was used for analysis (SigmaStat version 3.5, Point Richmond, CA). A p-value of <0.05 was considered statistically significant for all comparisons.

## Conclusions

The NLR is an easily collected and measured biomarker that correlates with clinical status in children with CF and may be indicative of conditions favorable to tissue damage in the CF lung. A breakpoint of 3 may be reasonable; however, larger prospective studies are needed to establish its utility as a biomarker that may be predictive of clinical outcome in this population.
